# Worsening or improving hypoalbuminemia during continuous renal replacement therapy is predictive of patient outcome: a single-center retrospective study

**DOI:** 10.1186/s40560-022-00620-9

**Published:** 2022-06-07

**Authors:** Harin Rhee, Gum Sook Jang, Sungmi Kim, Wanhee Lee, Hakeong Jeon, Da Woon Kim, Byung-min Ye, Hyo Jin Kim, Min Jeong Kim, Seo Rin Kim, Il Young Kim, Sang Heon Song, Eun Young Seong, Dong Won Lee, Soo Bong Lee

**Affiliations:** 1 Department of Internal Medicine, Pusan National University Hospital, Busan, Republic of Korea; 2 Divison of Nephrology, Biomedical Research Institute, Pusan National University Hospital, 305 Gudeok-ro, Seo-gu, Busan, 602-739 Republic of Korea; 3 Department of Internal Medicine, Pusan National University Yangsan Hospital, Yangsan, Republic of Korea; 4 Research Institute for Convergence of Biomedical Science and Technology, Pusan National University Yangsan Hospital, Yangsan, Republic of Korea

**Keywords:** Albumin, Acute kidney injury, Continuous renal replacement therapy, Hypoalbuminemia

## Abstract

**Background:**

Hypoalbuminemia at the initiation of continuous renal replacement therapy (CRRT) is a risk factor for poor patient outcomes. However, it is unknown whether the patterns of changes in serum albumin levels during CRRT can be used to predict patient outcomes.

**Methods:**

This retrospective study analyzed data that had been consecutively collected from January 2016 to December 2020 at the Third Affiliated Hospital. We included patients with acute kidney injury who received CRRT for ≥ 72 h. We divided the patients into four groups based on their serum albumin levels (albumin ≥ 3.0 g/dL or < 3.0 g/dL) at the initiation and termination of CRRT.

**Results:**

The 793 patients in this study were categorized into the following albumin groups: persistently low, 299 patients (37.7%); increasing, 85 patients (10.4%); decreasing, 195 patients (24.6%); and persistently high, 214 patients (27.1%). In-hospital mortality rates were highest in the persistently low and decreasing groups, followed by the increasing and persistently high groups. The hazard ratio for in-hospital mortality was 0.481 (0.340–0.680) in the increasing group compared to the persistently low group; it was 1.911 (1.394–2.620) in the decreasing group compared to the persistently high group. The length of ICU stay was 3.55 days longer in the persistently low group than in the persistently high group.

**Conclusions:**

Serum albumin levels changed during CRRT, and monitoring of patterns of change in serum albumin levels is useful for predicting in-hospital mortality and the length of ICU stay.

**Supplementary Information:**

The online version contains supplementary material available at 10.1186/s40560-022-00620-9.

## Background

Albumin is the most abundant protein in plasma; it has important roles in maintaining oncotic pressure, antibiotic delivery, anti-oxidation, and anticoagulation [1]. Critical illness alters the synthesis, degradation, and distribution of albumin between the intravascular and extravascular compartments [1–3], resulting in a dramatic decrease in serum albumin concentration from the early phase of illness.

Serum albumin levels change during the course of a disease. McCluskey et al. [4] reported that the serum albumin concentrations in non-survivors decrease rapidly during the first 24–48 h of ICU admission. Schreiber et al. [5] induced inflammation in rats and observed a decrease in albumin concentration for up to 36 h, followed by recovery to the baseline concentration.

Hypoalbuminemia has been reported in 73.9% of critically ill patients who require continuous renal replacement therapy (CRRT) [6, 7]. Several studies have reported that hypoalbuminemia at the initiation of CRRT is a prognostic marker for short- and long-term mortality [6, 8, 9]. However, little is known regarding the clinical importance of changes in serum albumin during CRRT.

The inflammatory response during a critical illness affects the life cycle and distribution of albumin between intravascular and extravascular compartments. We hypothesized that increasing or decreasing serum albumin levels reflect the resolution or maintenance of inflammation; thus, such patterns may provide information that can be used to predict patient survival. This study investigated changes in serum albumin during CRRT and their associations with outcomes in critically ill patients who required CRRT.

### Study design and population

This retrospective study enrolled consecutive patients who received CRRT between January 2016 and December 2020 at the Third Affiliated Hospital. We included patients aged ≥ 18 years who had acute kidney injury (AKI), with or without chronic kidney disease (CKD); we excluded patients with end-stage kidney disease who received maintenance dialysis treatment. Because we analyzed the associations between changes in serum albumin levels during CRRT and in-hospital mortality, we also excluded patients without serum albumin values, patients who received CRRT for < 72 h, and patients who remained in the hospital for > 100 days after CRRT.

### Data collection and definitions

The parameters included demographics, comorbidities, disease severity, and laboratory data at the initiation of CRRT. We obtained demographic and comorbidity data from medical records. Disease severity at the initiation of CRRT was assessed using the Sequential Organ Failure Assessment (SOFA) score. We reviewed the volume of urine within 6 h before the initiation of CRRT and defined oliguria as a mean urine output of < 0.5 mL/kg/h that was sustained for more than 6 h. The CRRT information included the number of days from ICU admission to the start of CRRT, the actual delivered dose, and the duration of CRRT. We extracted input and output information during CRRT from the electronic medical record and calculated the cumulative fluid balance using the following formula [10] [Σ (daily input (L) − daily output (L))/initial body weight (kg)] × 100. Reasons for termination of CRRT were grouped as follows: renal recovery, transition to conventional hemodialysis (HD), or inability to maintain CRRT because of low blood pressure/do not resuscitate (DNR) order/death. We collected longitudinal serum albumin data from the initiation to the termination of CRRT. Serum C-reactive protein (CRP) concentration was collected at the initiation of CRRT, 72 h after the initiation of CRRT, and at the termination of CRRT.

Based on the serum albumin level at the initiation of CRRT, we categorized the patients into initial normoalbuminemia (serum albumin ≥ 3.0 g/dL) and initial hypoalbuminemia (serum albumin < 3.0 g/dL) groups. Based on the serum albumin level at the termination of CRRT, the initial hypoalbuminemia group was further categorized into persistently low (serum albumin < 3.0 g/dL at CRRT termination) and increasing (serum albumin ≥ 3.0 g/dL at CRRT termination) groups; the initial normoalbuminemia group was further categorized into decreasing (serum albumin < 3.0 g/dL at CRRT termination) and persistently high (serum albumin ≥ 3.0 g/dL at CRRT termination) groups.

### CRRT operation

The decision to initiate CRRT was made at the discretion of the attending physician. After a patient had been advised to begin CRRT, a specialized CRRT team [11] initiated and managed dialysis. Vascular access was achieved through the internal jugular vein or femoral vein. CRRT was performed using the pre- and post-dilution continuous venovenous hemodiafiltration mode on a Baxter Prismaflex CRRT machine with an ST69 membrane. We used heparin as the primary anticoagulant; nafamostat mesylate was used as an alternative in patients with elevated risk of bleeding. The blood flow rate was 150 mL/min. PrismaSol was used for the dialysate and replacement solution. We prescribed a CRRT dose of 35 mL/kg/h with hemodialysis and hemofiltration at 1:1. The actual delivered dose was calculated based on the effluent flow rate after correction for the prediluted percentage.

Considering the SAFE study results [12, 13], we did not routinely infuse commercially available albumin for the correction of hypoalbuminemia during CRRT. The decision to terminate CRRT was made by the nephrologist on the CRRT team when the patient’s blood pressure had recovered without the assistance of vasopressors (in this case, we changed the dialysis mode to conventional hemodialysis if further renal replacement therapy was needed) or when there was a trend of increased urine output [14]. We also stopped CRRT in cases of patients with very low blood pressure (usually mean arterial pressure < 60 mmHg) that did not respond to the full doses of multiple vasopressors or inotropes and maintaining CRRT was expected to cause shock.

### Outcomes

The primary outcome was in-hospital mortality. The secondary outcomes were the lengths of ICU and hospital stays.

### Statistical analysis

The normality of the data was verified using the Kolmogorov–Smirnov test. Continuous variables are expressed as median and interquartile range or mean ± standard deviation, as appropriate. Differences in parameters among the four albumin groups were compared using one-way analysis of variance; post hoc analyses were corrected using the Bonferroni method. Categorical variables are expressed as percentages; differences in proportions were compared using the Chi-squared test. Longitudinal changes in serum albumin levels were compared via repeated-measures analysis. Survival curves were plotted using the Kaplan–Meier method and analyzed using log-rank tests to show the un-adjusted effect of each albumin group on in-hospital mortality.

The effects of changes in serum albumin on in-hospital mortality were analyzed using univariable and multivariable Cox proportional hazards models. The full model was adjusted for age, sex, body mass index (BMI), diabetes, liver cirrhosis, chronic obstructive pulmonary disease (COPD), CKD, cancer, sepsis, hemoglobin, SOFA score, number of days from ICU to CRRT, actual delivered CRRT dose, and CRRT duration. The final model was determined using backward model selection based on the Wald test, with a 0.2 threshold for predictors (e.g., age, COPD, CKD, SOFA score, and actual delivered CRRT dose). We also included BMI and sepsis in the final model, regardless of the Wald test result, because BMI (a basic nutrition factor) [15–17] and sepsis [2, 18] have critical effects on albumin that influence in-hospital mortality.

Estimates of the effects of changes in albumin on the length of ICU or hospital stay were analyzed using a multiple linear regression model adjusted for age, SOFA score, and CRRT duration. All statistical tests were two-sided, and *p*-values < 0.05 were considered statistically significant. Data were analyzed and plotted using IBM SPSS software (v. 28.0; SPSS Inc., Chicago, IL, USA) or SigmaPlot (v.10.0; Systat Software Inc., San Jose, CA, USA).

## Results

**Fig. 1:**
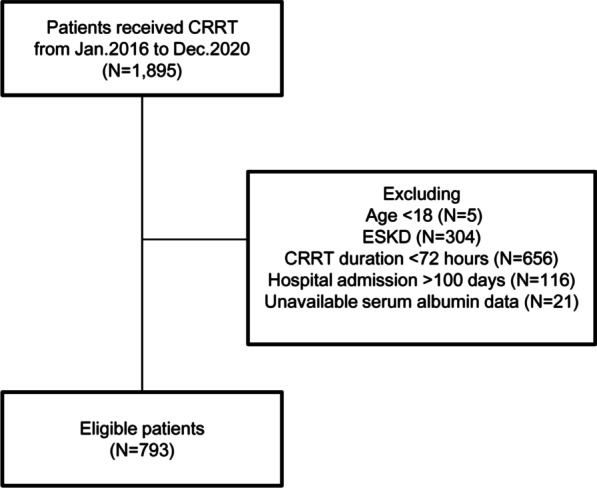
Flow diagram of the study population

In total, 1895 patients received CRRT in our hospital between January 2016 and December 2020. We excluded 5 patients who were < 18 years of age, 304 patients who had end-stage kidney disease, 656 patients who received CRRT for < 72 h, 116 patients who remained in the hospital for > 100 days, and 21 patients who had no serum albumin data during CRRT. Thus, we included 793 patients in the analysis (Fig. 1).

The median age of the patients was 67.5 (56–77) years; 63.1% of the patients were men, and the mean BMI was 24.3 ± 12.9 kg/m^2^. The patients had a median of 1 (1–2) comorbidity; diabetes (41.8%), hypertension (51.9%), and congestive heart failure (26.6%) were the most common comorbidities. Liver cirrhosis was observed in 11.2% of the patients, and sepsis was diagnosed in 23.7% of the patients. The mean SOFA score at the initiation of CRRT was 10.34 ± 3.74.

**Fig. 2:**
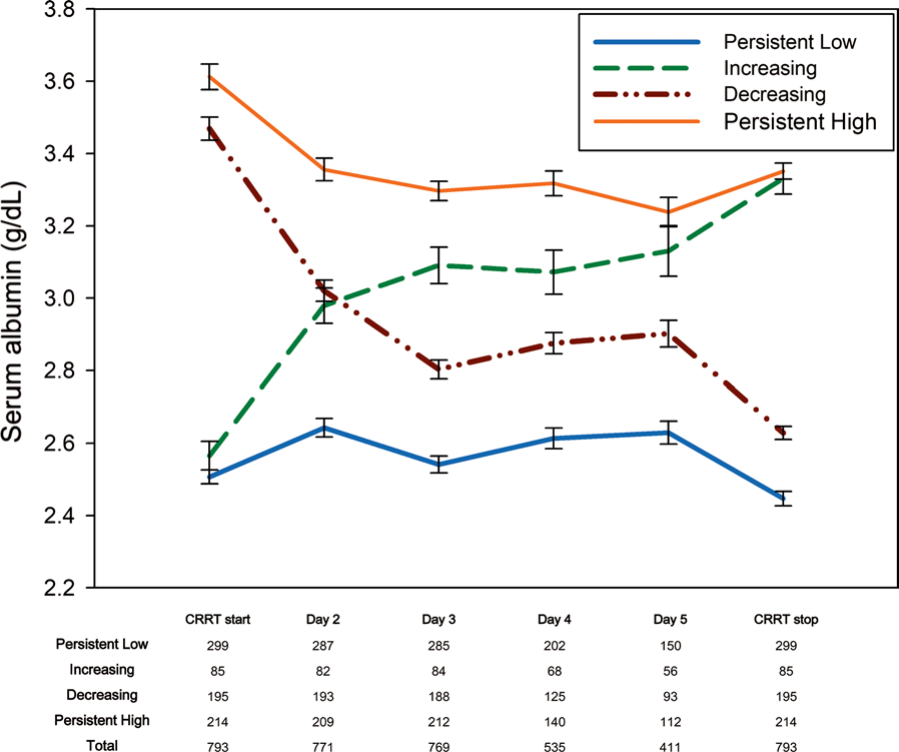
Changes in serum albumin level during continuous renal replacement therapy in the four albumin groups: orange, persistently high; green, increasing; purple, decreasing; blue, persistently low. Numbers at the bottom of the figure indicate the number of data available for each group on each day

The mean serum albumin level at the initiation of CRRT was 3.05 ± 0.67 g/dL. Serum albumin levels changed during the median of 5 (3–8) days of CRRT. Among the 793 included patients, serum albumin levels were persistently low in 299 (37.7%), increased in 85 (10.4%), decreased in 195 (24.6%), and persistently high in 214 (27.0%). Changes in the serum albumin level in each group are plotted in Fig. 2. In the decreasing and increasing groups, serum albumin levels started to change on the second day of CRRT, and on the third day (day 3) of CRRT, mean serum albumin levels were highest in the persistently high, followed by the increasing, decreasing, and the persistently low group. At the end of CRRT, the mean serum albumin level in the increasing group was similar to that in the persistently high group.

Thirty-seven of 793 (4.7%) patients received intravenous albumin during CRRT; 15 (5.0%) among the persistently low, 5 (5.9%) among the increasing, 9 (4.6%) among the decreasing, and 8 (3.7%) among the persistently high albumin group. No difference in proportion was detected.

### Associations of patient characteristics and disease course with changes in serum albumin levels

In the initial normoalbuminemia group, congestive heart failure, COPD, and CKD were more common, whereas sepsis and the use of vasopressors were less common than in the initial hypoalbuminemia group. Mean arterial pressure was higher and SOFA score was lower in the initial normoalbuminemia group at the initiation of CRRT (Additional file 1: Table S1).

Among the patients with initial hypoalbuminemia, the increasing group received CRRT longer [8 (5–13.3) days] than did the persistently low group [6 (4–11) days, *p* < 0.001]. The initial laboratory findings and disease severity did not differ between the two groups, except for the lower CRP levels in the increasing albumin group. A greater proportion of patients in the increasing group transitioned to conventional HD (21.2% vs. 12.4, *p* = 0.041), while a smaller proportion of patients terminated CRRT because of low BP/DNR/death (41.2% vs. 53.2%, *p* = 0.049), compared to the persistently low group (Table 1).

**Table 1:** Baseline characteristics of patients by the serum albumin change pattern

	Alb < 3.0 g/dL at CRRT start		Alb ≥ 3.0 g/dL at CRRT start	
	Persistent low (*N* = 299)	Increasing (*N* = 85)	Decreasing (*N* = 195)	Persistent high (*N* = 214)
Demographics				
Age, year	66 (55–75)	63.5 (52.5, 75.3)	70 (61, 77.5)^†^	64 (52, 75)
Male, %	67.9	64.3	66.7	60.3
BMI, kg/m^2^	23.6 ± 4.7	24.3 ± 4.2	24.0 ± 4.0	24.0 ± 4.3
Comorbidities				
Diabetes, %	40.8	34.8	42.9	40.3
Hypertension, %	42.7	39.4	53.2	50.0
Liver cirrhosis, %	12.2	25.8*	8.7	11.1
CHF, %	22.5	22.7	34.9	33.3
COPD %	4.2	6.1	11.9	6.9
CKD, %	8.9	13.6	19.8	17.4
Cancer, %	19.1	12.9	21.0^†^	11.7
Disease severity				
Sepsis, %	36.2	22.9	18.6^†^	6.2
Anuria, %	59.2	59.3	52.6^†^	63.3
MAP, mmHg	79.1 ± 14.7	80.6 ± 16.6	83.9 ± 18.2	83.2 ± 15.9
Vasopressors, %	68.2	68.6	60.5^†^	49.3
Ventilator, %	61.1	68.6	60.5	58.9
SOFA score	11.4 ± 3.7	11.1 ± 3.3	9.9 ± 3.5	9.0 ± 3.4
Laboratory data				
WBC, 10^3^/μL	14.2 ± 10.9	13.2 ± 11.4	13.5 ± 10.6	13.5 ± 11.8
Platelet, 10^3^/μL	136.9 ± 116.5	122.2 ± 86.4*	162.1 ± 99.7	150.3 ± 94.3
Hemoglobin, g/dL	9.9 ± 5.8	9.6 ± 5.1	11.4 ± 3.2	10.7 ± 2.7
Total protein, g/dL	5.0 ± 1.1	4.8 ± 0.9	6.1 ± 1.4	5.9 ± 1.1
Albumin, g/dL	2.5 ± 0.4	2.6 ± 0.3	3.5 ± 0.5^†^	3.6 ± 0.5
BUN, mg/dL	62.5 ± 36.2	54.7 ± 35.3	57.1 ± 38.5	52.4 ± 34.5
Creatinine, mg/dL	3.5 ± 2.4	3.2 ± 2.5	4.5 ± 8.8	3.2 ± 2.6
Sodium, mmol/L	136.4 ± 8.2	137.4 ± 7.8	136.5 ± 8.3	137.8 ± 7.7
Potassium, mmol/L	4.4 ± 0.9	4.3 ± 0.9	5.8 ± 12.4	4.6 ± 1.0
CRP, mg/L	13.6 ± 11.3	7.7 ± 8.7*	9.3 ± 10.1^†^	5.3 ± 7.0
PT, INR	1.8 ± 1.3	1.8 ± 0.9	1.4 ± 0.4^†^	1.8 ± 1.7
CRRT operation				
ICU to RRT, days	1 (0, 4)	1 (1, 6)	1 (0, 2)	0 (0, 1)
Duration, days	6 (4, 11)	8 (5, 13.3)*	6 (5, 8)	6 (5, 10)
Delivered dose, ml/kg/h	34.1 ± 5.3	34.9 ± 3.9	33.7 ± 4.9	35.3 ± 5.6
IV albumin infusion,%	15 (5.0)	5 (5.9)	9 (4.6)	8 (3.7)
Reasons for CRRT stop				
Kidney recovery	34.4	37.6	46.7	52.8
Transition to HD	12.4	21.2*	13.8	17.3
Unable to maintain CRRT (low BP/DNR/death)	53.2	41.2*	39.5^†^	29.9

*p < 0.05, compared to persistent low, ^†^*p* < 0.05 compared to persistent high

*Alb* albumin, *CRRT* continuous renal replacement therapy, *BMI* body mass index, *CHF* congestive heart failure, *COPD* chronic obstructive pulmonary disease, *CKD* chronic kidney disease, *MAP* mean arterial pressure, *SOFA* Sequential Organ Failure Assessment, *WBC* white blood cell, *BUN* blood urea nitrogen, *PT* prothrombin time, *ICU* intensive care unit, *RRT* renal replacement therapy

Among the patients with initial normoalbuminemia, patients in the decreasing group were older [70 (61–77.5) years vs. 64 (52–75) years, *p* = 0.002] and were more likely to have cancer (21% vs. 11.7%, *p* = 0.010) than were patients in the persistently high group. Sepsis was more common (18.6% vs. 6.2%, *p* < 0.001), vasopressors were more frequently required (60.5% vs. 49.3%, *p* = 0.004), and CRP levels were higher in the decreasing group than in the persistently high group. The proportion of patients who terminated CRRT because of low BP/DNR/death was higher (39.5% vs. 29.9%, *p* = 0.042) in the decreasing group (Table 1).

### In-hospital mortality according to changes in serum albumin levels

**Fig. 3:**
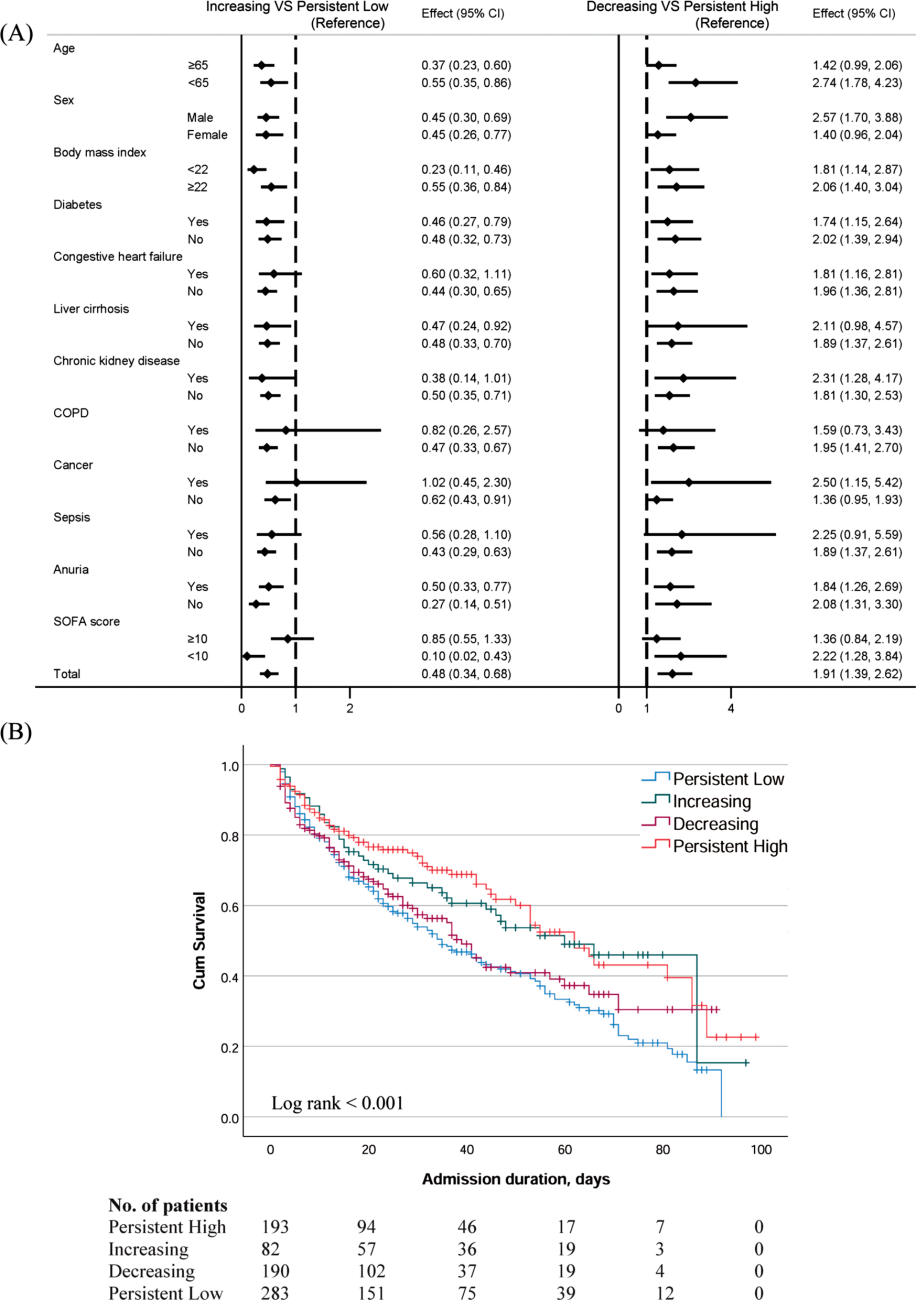
**A** Hazard ratios for in-hospital mortality of the increasing group compared to the persistently low group (left), and the decreasing group compared to the persistently high group (right) stratified by age, sex, body mass index, diabetes, congestive heart failure, liver cirrhosis, chronic kidney disease, chronic obstructive pulmonary disease, sepsis, anuria, and SOFA score. **B** Kaplan–Meier survival analysis results of the four albumin change groups; orange, persistently high; green, increasing; purple, decreasing; blue, persistently low

In total, 378 (47.9%) of the 789 patients died during their hospital stay. The in-hospital mortality rate was higher in the initial hypoalbuminemia group than in the initial normoalbuminemia group (Additional file 1: Fig. S1). Among the patients with initial hypoalbuminemia, the hazard ratio (HR) for in-hospital mortality was significantly lower in the increasing group than in the persistently low group (Fig. 3A, left); among the patients with initial normoalbuminemia, the HR for in-hospital mortality was significantly higher in the decreasing group than in the persistently high group (Fig. 3A, right). In the subgroup of patients with sepsis, cancer, COPD, or a higher SOFA score (SOFA score > 10), the HRs did not significantly differ between the increasing and persistently low groups or the decreasing and persistently high groups. Analysis of all patients showed that the in-hospital mortality rates were highest in the persistently low and decreasing groups, followed by the increasing and persistently high groups (log-rank *p* < 0.01) (Fig. 3B).

Table 2 shows the HRs of the albumin groups for prediction of in-hospital mortality after adjustments for age, BMI, COPD, CKD, sepsis, SOFA score, and CRRT dose. The HRs for in-hospital mortality were 0.571 (0.340–0.958) in the increasing group and 0.565 (0.353–0.903) in the persistently high group compared to the persistently low group. The HR of the decreasing group for in-hospital mortality was 0.722 (0.455–1.146), which was similar to the HR of the persistently low group. Similar results were observed in the fully adjusted model (Additional file 1: Table S2). In addition to the change in serum albumin, old age [HR 1.014 (1.002–1.027)] and a higher SOFA score [HR 1.089 (1.036–1.45)] were significant predictors of in-hospital mortality (Table 2).

**Table 2:** Multi-variable Cox regression analysis results for predicting in-hospital mortality

	HR (95% CI)	*p*-value
Model I		
Albumin changing pattern		
Persistent low	Reference	
Increasing	0.571 (0.340, 0.958)	0.034
Decreasing	0.722 (0.455, 1.146)	0.167
Persistent high	0.565 (0.353, 0.903)	0.017
Age, year	1.014 (1.002, 1.027)	0.023
SOFA score	1.089 (1.036, 1.455)	< 0.001
Model II		
Serum albumin at Day 3, g/dL	0.532 (0.377, 0.751)	< 0.001
Age, year	1.017 (1.006, 1.029)	0.004
COPD	1.887 (1.104, 3.226)	0.020
SOFA score	1.088 (1.042, 1.137)	< 0.001

Final model: adjusted with age, BMI, COPD, CKD, sepsis, SOFA score and CRRT delivered dose

*SOFA* Sequential Organ Failure Assessment, *BMI* body mass index, *COPD* chronic obstructive pulmonary disease, *CKD* chronic kidney disease, *CRRT* continuous renal replacement therapy

As day 3 serum albumin levels clearly separated the four albumin groups, we further tested its performance in predicting in-hospital mortality. After adjusting for the parameters listed above, lower serum albumin on day 3 (HR 0.532; 0.377, 0.751) was significant for predicting in-hospital mortality (Table 2).

### Lengths of ICU and hospital stays according to changes in serum albumin levels

The median length of ICU stay was 11 (6–20) days; it was longer in the decreasing group [16 (10–30) days] than in the increasing [10 (6–17) days] or persistently high [9.5 (6–15) days] groups (*p* < 0.001) (Additional file 1: Table S3). In the multivariable linear regression model adjusted for age, SOFA score, and CRRT duration, the median length of ICU stay was 3.55 (0.49–6.60) days longer in the persistently low group than in the persistently high group (*p* = 0.023) (Table 3).

**Table 3:** Impact of the albumin change pattern on length of stay at ICU or hospital

	Differences in admission duration Mean (95% CI), days	*P*-value
LOS at ICU, days		
Albumin change pattern		
Persistent Low	3.55 (0.49, 6.60)	0.023
Increasing	2.55 (− 1.51, 6.61)	0.218
Decreasing	1.78 (− 1.56, 5.11)	0.295
Persistent High	Reference	
CRRT duration, days	1.01 (0.83, 1.19)	< 0.001
LOS at hospital, days		
Albumin change pattern		
Persistent Low	2.69 (− 2.60, 7.97)	0.318
Increasing	5.43 (− 1.76, 12.6)	0.138
Decreasing	− 0.20 (− 6.08, 5.68)	0.946
Persistent High	Reference	
Age, year	− 0.17 (− 0.31, − 0.03)	0.015
CRRT duration, days	0.90 (0.56, 1.21)	< 0.001

Final model: adjusted with age, SOFA score, and CRRT duration

*ICU* intensive care unit, *CRRT* continuous renal replacement therapy, *SOFA* Sequential Organ Failure Assessment, *LOS* length of stay

The median length of hospital stay was 22 (12–42) days; it was longer in the decreasing group [69 (16–58) days] than in the increasing group [21 (10–35) days] (Additional file 1: Table S3). However, the multivariable linear regression model adjusted for age, SOFA score, and CRRT duration revealed no differences in the length of hospital stay according to changes in serum albumin levels. In this study, the length of hospital stay was affected by age (mean difference, − 0.17 (− 0.31 to − 0.03) days) and CRRT duration (mean difference, 0.90 (0.56–1.21) days) (Table 3).

In the multivariable linear regression model, each 1 g/dL decrease in serum albumin level on day 3 predicted 3.96 (0.20, 7.89) days longer duration of hospital stay, whereas it was not predictive for the length of the ICU stay (Additional file 1: Table S4).

### Changes in volume status during CRRT according to the serum albumin group

**Fig. 4:**
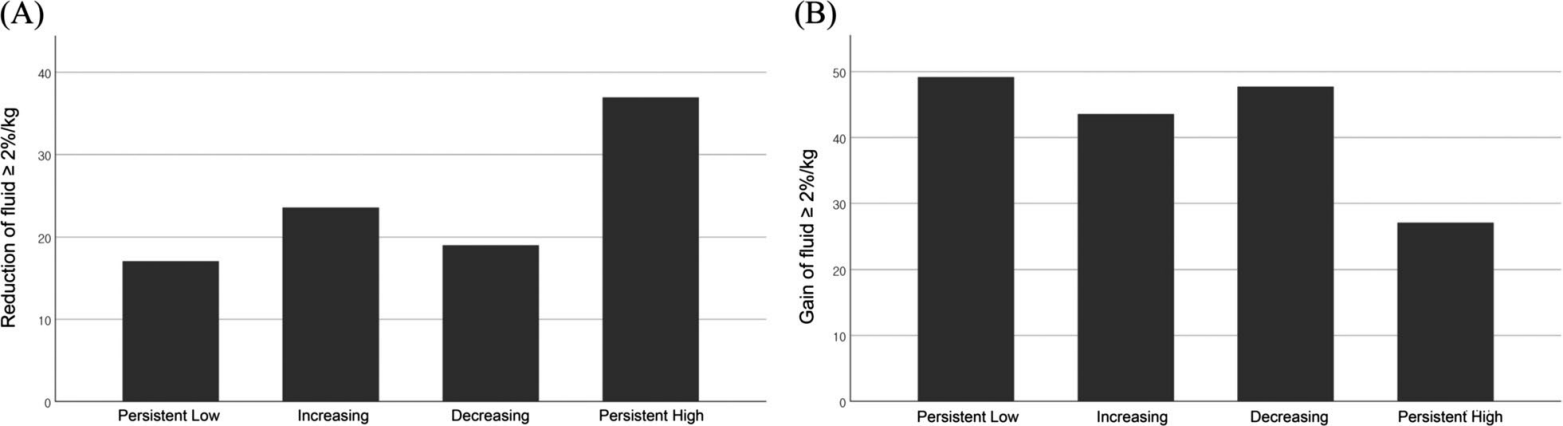
**A** The proportion of patients with a fluid reduction more than 2% of their initial body weight in the four albumin groups. **B** The proportion of patients with a fluid gain of more than 2% of initial body weight in the four albumin groups

During the median 5 (3–8) days of CRRT, mean total input was 24.3 ± 34.8 L, the mean total output was 21.6 ± 24.9 L, and the mean total fluid balance was 2.1 ± 6.5L, which was 2.9 ± 9.8% of initial body weight. The mean changes in volume status per body weight in each group were 5.6 ± 9.5%/kg in the persistently low, 3.2 ± 9.2%/kg in the increasing, 3.2 ± 10.2%/kg in the decreasing, and − 0.2 ± 9.2%/kg in the persistently high group (Additional file 1: Table S5). The proportion of patients with more than a 2%/kg volume decrease was highest in the persistently high group (43.2%) followed by the increasing (27.0%), decreasing (22.7%), and persistently low groups (20.3%), whereas the proportion of patients with more than 2%/kg added volume was highest in the persistently low (58.6%) and decreasing (57.1%) groups followed by the increasing (50%) and persistently high (31.7%) albumin groups (Fig. 4).

### Changes in CRP during CRRT according to the serum albumin group

**Fig. 5:**
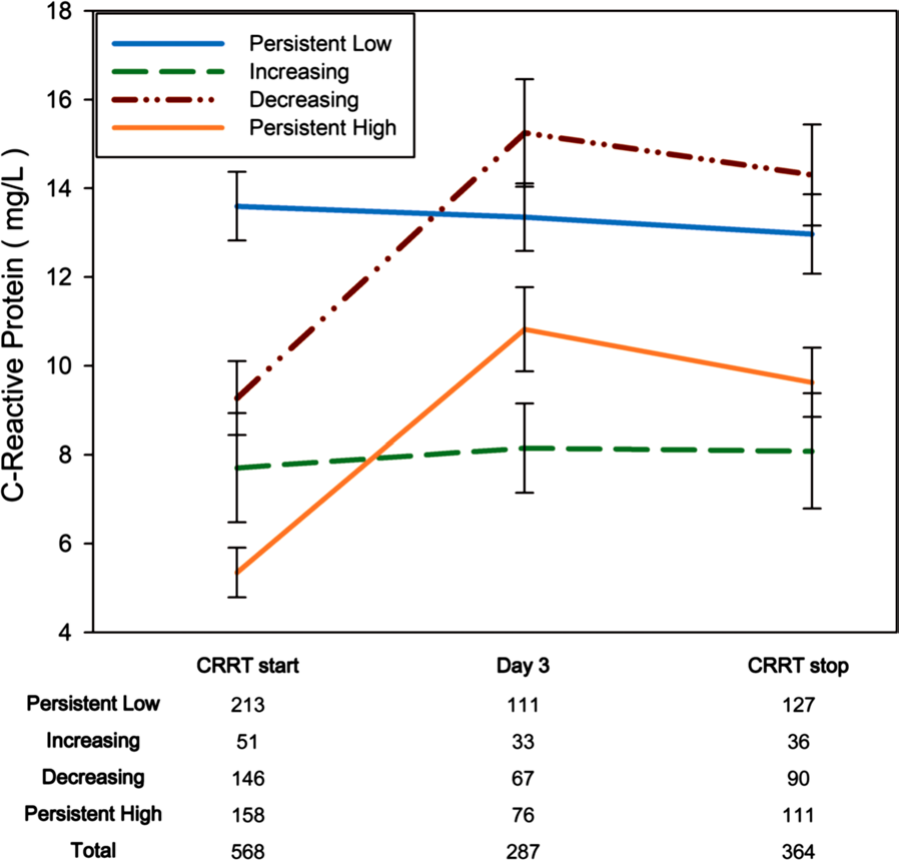
Changes in serum CRP levels during continuous renal replacement therapy in the four albumin groups: orange, persistently high; green, increasing; purple, decreasing; blue, persistently low. Numbers at the bottom of the figure show the number of data available for each group and each day

Information for CRP was available in the 72.3%, 36.7%, and 46.6% of the groups at the start of CRRT, CRRT day 3, and upon stopping CRRT. As shown in Fig. 5, serum CRP levels were persistently high in the persistent low albumin group and significantly increased during CRRT in the decreasing albumin group. CRP levels were persistently low in the increasing albumin group, which was comparable to those in the persistently high albumin group.

## Discussion

Serum albumin level changed rapidly beginning on day 2 of CRRT; at the start of CRRT, the mean serum albumin level in the decreasing group was significantly higher than that in the increasing group, whereas the two groups had similar levels on day 2, which reversed on day 3. The patterns of changes in serum albumin levels during a median of 5 (3–8) days of CRRT were predictive of in-hospital mortality and the length of ICU stay. The in-hospital mortality rate was lower in the increasing group than in the persistently low group; it was higher in the decreasing group than in the persistently high group. ICU stays were significantly longer in the persistently low group than in the persistently high group. We also observed that the serum albumin level on day 3 of CRRT was predictive of in-hospital mortality and the length of hospital stay. To our knowledge, this is the first report to show diverse changes in serum albumin levels during CRRT and their associations with patient outcomes.

Several studies have reported the effect of initial hypoalbuminemia on mortality in AKI patients who required CRRT. Moon et al. [8] retrospectively reviewed 1581 AKI patients who received CRRT and classified them into three groups based on tertiles of serum albumin level at the beginning of CRRT; they compared 1-week, 1-month, and 6-month all-cause mortality rates. The 1-week, 1-month, and 6-month mortality rates were 1.3- to 1.4-fold higher in the second tertile (mean serum albumin level, 2.7 ± 0.1 g/dL) and 2.7- to 3.2-fold higher in the first tertile (mean serum albumin level, 2.1 ± 0.3 g/dL) than in the third tertile (mean serum albumin level, 3.4 ± 0.4 g/dL).

Zheng et al. [9] reviewed 837 AKI patients who received CRRT; they analyzed the effects of initial hypoalbuminemia on short- and long-term mortality rates. In multivariate analysis, the HRs of initial hypoalbuminemia for 28-day and 90-day mortality were 0.63 (0.50–0.80) and 0.63 (0.51–0.78), respectively. The association between hypoalbuminemia and mortality was verified by Lv et al. [6] in a retrospective review of 1132 AKI patients who required CRRT; their results showed HRs for 28-day mortality and 90-day mortality of 0.92 (0.89–0.95) and 0.92 (0.89–0.96), respectively.

Consistent with previous reports, we found that initial hypoalbuminemia influenced in-hospital mortality. The patient characteristics and initial disease severities significantly differed according to the initial serum albumin level; in the normoalbuminemia group, the proportion of comorbidities was higher, disease severity was milder, and the time from ICU to CRRT was shorter. Similar trends were observed in previous studies. In Lv et al. [6], the proportions of myocardial infarction and congestive heart failure were higher, while the mean SOFA score was lower, in the group with serum albumin ≥ 3 g/dL than in the group with serum albumin < 3 g/dL. Moon et al. [8] showed that the proportion of patients with diabetes was higher, while the mean APACHE II score was lower, in the third tertile of albumin compared to the other tertiles. We speculate that patients with initial normoalbuminemia received CRRT earlier in the course of acute illness, possibly because of a higher comorbidity burden. Although baseline nutritional status [19, 20], liver cirrhosis [21], and chronic cancer [22] may have extensive effects on the baseline serum albumin level at the initiation of CRRT, the baseline serum albumin level may also reflect the duration of inflammation before the initiation of CRRT [1, 17].

Albumin is synthesized in the liver, and the rate of synthesis varies depending on nutritional intake [1]. Therefore, patients with liver cirrhosis or who are malnourished tend to have lower serum albumin levels. Patients with cancer also tend to have lower serum albumin levels due to the hepatotoxic effects of chemotherapeutic agents or chronic malnutrition. As critical illness affects the synthesis and degradation of albumin [1], the pattern of change in serum albumin and the clinical significance may differ in patients with liver cirrhosis or cancer with critical illness. We separately analyzed the performance of the changes in serum albumin in patients with/without these conditions. In our data, lower BMI or having liver cirrhosis did not change the result, whereas having a cancer attenuated the performance of the albumin level for predicting mortality. It is probable that the characteristics of the cancer (type, aggressiveness) or treatment response to each cancer may be stronger factors affecting patient outcome than serum albumin. Therefore, patterns of change in serum albumin should be interpreted cautiously when predicting the outcomes of critically ill patients with cancer.

Critical illness alters the distribution of albumin between the intravascular and extravascular compartments; this alteration is caused by increased capillary leakage [1, 2]. Importantly, endothelial barrier dysfunction leads to capillary leakage, loss of protein, and substantial fluid entry into the interstitial space. Mediators of capillary leakage include endotoxins from Gram-negative bacteria [23, 24], tumor necrosis factor-alpha [25], interleukin-6 [26], leukotrienes [27], prostaglandins [27], bradykinins, histamine [24], and macrophage inflammatory protein 1 alpha [28]; all of these components can be controlled by appropriate treatment. Thus, we hypothesized that changes in serum albumin levels during the course of disease would reflect the maintenance or resolution of the inflammatory process. The changes in serum CRP in our data support the hypothesis. Serum CRP levels were persistently high in the persistently low albumin group and increased with time in the decreasing albumin group, which implied un-resolving or worsening of the inflammation. CRP levels were relatively low in the increasing or persistently high albumin groups.

The patient outcomes were additional indirect evidence supporting the hypothesis. A greater proportion of patients transitioned to conventional HD and fewer patients terminated CRRT because of low BP/DNR/death in the increasing group. Patient outcomes were worse in the decreasing group than in the persistently high group; in the decreasing group, greater proportions of patients terminated CRRT because of low BP/DNR/death, remained in the hospital longer, and died in the hospital. Compared to the persistently high group, patients in the decreasing group were older, more likely to have cancer, more likely to have sepsis, and more frequently required vasopressors; thus, a greater burden of acute illness was present in patients who had limited capacity to tolerate that illness. Considering the demand–capacity balance during the disease course [29], the changes in serum albumin during CRRT might be a reflection of this process.

Volume overload is a risk factor for mortality in AKI patients [10, 30–32], and adequate fluid removal ameliorates the toxic effect [10, 30]. Because hypervolemia results in dilutional hypoalbuminemia [33], we compared total fluid balance of each albumin group during CRRT. As expected, fluid removal was best in the persistently high followed by the increasing group, whereas a greater proportion of patients gained fluid after CRRT in the decreasing and persistently low albumin groups. Although the exact contribution of fluid volume to serum albumin concentration was not analyzed due to the diversity of the fluid registered, the pattern of volume change after CRRT supported the possible role of dilutional hypoalbuminemia in the short-term serum albumin changes during CRRT.

The benefit of a commercially available albumin infusion could not be investigated in this study. Because the SAFE study [12, 13] showed no additional benefit from an albumin infusion compared to normal saline in critically ill patients [13], we did not routinely infuse albumin. In our study, only 4.3% of the patients received an albumin infusion during the CRRT. Therefore, we do not know whether the improvement of hypoalbuminemia led to direct improvement of patient outcomes. We suspect that changes in serum albumin level during CRRT constituted a marker for changes in inflammation or volume status.

The limitations of this study should be discussed. First, due to the retrospective nature of this study, some data were missing, such as the serum CRP levels were missing in 27.6% of the patients. Second, we did not reveal a direct contribution of inflammation or volume status on short-term serum albumin changes due to the lack of inflammatory markers other than CRP, and the diversity of fluid registered during CRRT. Further well-designed prospective studies will be needed to reveal this issue. A notable strength of this study is that it shows the clinical usefulness of monitoring serum albumin during CRRT, which has not been previously reported.

## Conclusions

An increase in the serum albumin level at the termination of CRRT is predictive of lower in-hospital mortality compared to a persistently low albumin level. A decrease in the serum albumin level during CRRT, which is more common in older and septic patients, is predictive of higher in-hospital mortality, compared to a persistently high serum albumin level.

## Supplementary Information

**Additional file 1: Table S1.** Comparison of the baseline characteristics by serum albumin level at the initiation of continuous renal replacement therapy. **Figure S1.** Kaplan–Meier survival analysis for in-hospital mortality by serum albumin level at the initiation of continuous renal replacement therapy. **Table S2.** Cox regression analysis results of the effects of changes in serum albumin level on in-hospital mortality. **Table S3.** Comparison of patient outcomes according to changes in serum albumin level. **Table S4.** Effect of day 3 serum albumin on the length of stay in the ICU or hospital. **Table S5.** Changes in fluid balance during CRRT based on the pattern of the change in serum albumin.

## Data Availability

The datasets used and/or analyzed during this study are available from the corresponding author upon reasonable request.

## Abbreviations

AKI: Acute kidney injury
BP: Blood pressure
CKD: Chronic kidney disease
CRP: C-reactive protein
CRRT: Continuous renal replacement therapy
DNR: Do not resuscitate
HD: Hemodialysis
HR: Hazard ratio
ICU: Intensive care unit
SOFA: Sequential Organ Failure Assessment
