# Supplementary material for: Worsening or improving hypoalbuminemia during continuous renal replacement therapy is predictive of patient outcome: a single-center retrospective study
Source: J Intensive Care. 2022 Jun 7;10:25. doi: 10.1186/s40560-022-00620-9 (PMC9171968; doi:10.1186/s40560-022-00620-9)
Supplement: Supplementary file 1 — Additional file 1: Table S1. Comparison of the baseline characteristics by serum albumin level at the initiation of continuous renal replacement therapy. Figure S1. Kaplan–Meier survival analysis for in-hospital mortality by serum albumin level at the initiation of continuous renal replacement therapy. Table S2. Cox regression analysis results of the effects of changes in serum albumin level on in-hospital mortality. Table S3. Comparison of patient outcomes according to changes in serum albumin level. Table S4. Effect of day 3 serum albumin on the length of stay in the ICU or hospital. Table S5. Changes in fluid balance during CRRT based on the pattern of the change in serum albumin. [file 40560_2022_620_MOESM1_ESM.pdf]

Supplementary Table 1. Comparison of the baseline characteristics by serum albumin level at the initiation of continuous renal replacement therapy

	Serum albumin <3.0g/dL (N=384)	Serum albumin ≥3.0g/dL (N=409)	P-value
**Demographics**			
Age, year	67(56-76)	68(57, 77)	0.382
Male, %	64.2	62.6	0.633
BMI, kg/m2	24.6±18.1	24.0±4.4	0.534
**Comorbidities**			
Diabetes, %	41.6	42.1	0.892
Hypertension, %	44.0	50.7	0.060
Liver Cirrhosis, %	14.7	7.9	0.003
CHF, %	22.4	30.4	0.011
COPD %	4.5	9.9	0.004
CKD, %	10.1	18.3	0.001
Cancer, %	17.7	16.1	0.555
**Disease Severity**			
Sepsis, %	74.7	51.3	<0.001
Anuria, %	60.2	58.3	0.618
MAP, mmHg	79.3±15.1	82.2±16.6	0.011
Vasopressors, %	70.0	56.2	<0.001
Ventilator, %	62.3	57.7	0.187
SOFA score	11.5±3.6	9.2±3.5	<0.001
**Laboratory data**			
WBC, 10E3/uL	13.8±11.2	13.8±11.2	0.965
Platelet, 10E3/uL	134.3±108.4	164.1±95.9	<0.001
Hemoglobin, g/dL	9.8±4.6	11.1±2.9	<0.001
Total protein, g/dL	5.3±6.9	6.1±3.5	0.032
Albumin, g/dL	2.5±0.3	3.5±0.5	<0.001
BUN, mg/dL	60.0±35.2	55.7±38.2	0.048
Creatinine, mg/dL	3.3±2.6	3.8±5.1	0.080
Sodium, mmol/L	136.9±7.9	136.8±7.2	0.441
Potassium, mmol/L	4.4±0.9	5.0±6.3	0.027
CRP, mg/L	12.5±11.1	7.2±8.8	<0.001
PT, INR	1.8±1.2	1.7±1.3	0.079
**CRRT operation**			
ICU to RRT, days	1(0, 4)	0(0, 2)	<0.001
Duration, days	5(3.3, 9)	5(3, 7)	0.004
Delivered dose, ml/kg/hr	34.4±5.6	34.7±5.9	0.546

Abbreviations: Alb, albumin; CRRT, continuous renal replacement therapy; BMI, body mass index; CHF, congestive heart failure; COPD, chronic obstructive pulmonary disease; CKD, chronic kidney disease; MAP, mean arterial pressure; SOFA, sequential organ failure assessment; WBC, white blood cell; BUN, blood urea nitrogen;

PT, prothrombin time; ICU, intensive care unit; RRT, renal replacement therapy

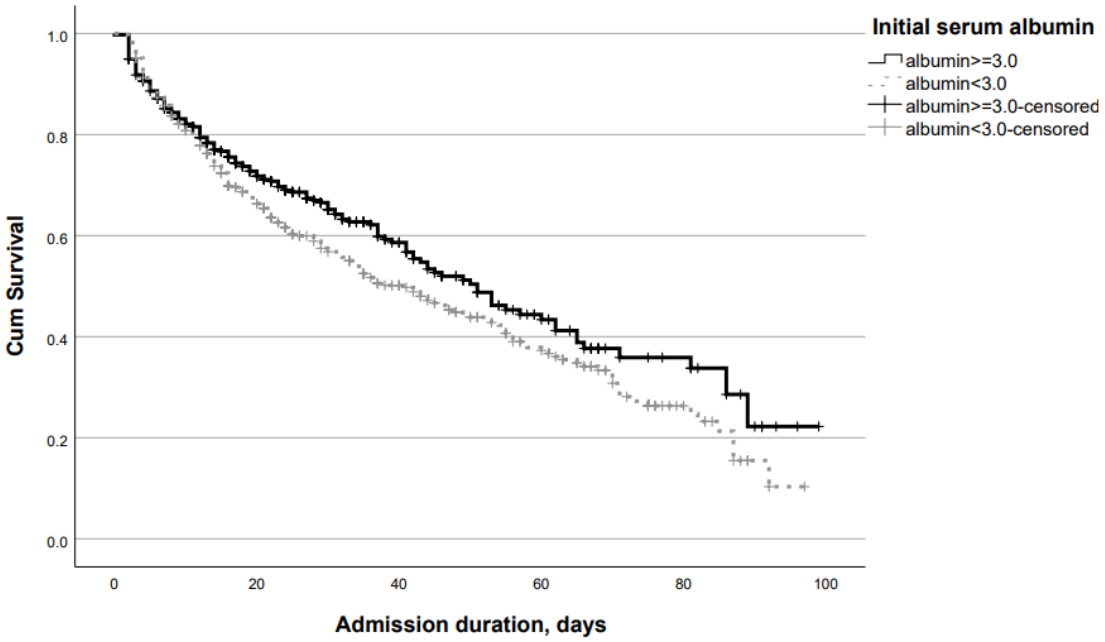

Supplementary Figure 1. Kaplan-Meier survival analysis for in-hospital mortality by serum albumin level at the initiation of continuous renal replacement therapy

Supplementary Table 2. Cox regression analysis results of the effects of changes in serum albumin level on in-hospital mortality

	HR (95%CI)	P-value
Unadjusted		
Persistent Low	Reference	
Increasing	0.647(0.460, 0.910)	0.012
Decreasing	0.887(0.687, 1.144)	0.356
Persistent High	0.584(0.443, 0.769)	<0.001
Full model		
Persistent Low	Reference	
Increasing	0.513(0.299, 0.881)	0.016
Decreasing	0.731(0.455, 1.173)	0.194
Persistent High	0.556(0.345, 0.897)	0.016
Final model		
Persistent Low	Reference	
Increasing	0.571(0.340, 0.958)	0.034
Decreasing	0.722(0.455, 1.146)	0.167
Persistent High	0.565(0.353, 0.903)	0.017

Full model: adjusted with age, sex, body mass index, diabetes, liver cirrhosis, COPD, CKD, cancer, sepsis, hemoglobin, SOFA score, days from ICU to CRRT, CRRT delivered dose and CRRT duration

Final model : adjusted with age, BMI, COPD, CKD, sepsis, SOFA score and CRRT delivered dose

Supplementary Table 3. Comparison of patient outcomes according to changes in serum albumin level.

	Persistent Low (N=296)	Decreasing (N=85)	Increasing (N=195)	Persistent High(N=213)	P
Death, N(%)	175(59.1)ǂ	41(48.2)ǂ	90(46.2)	72(33.8)	<0.001
Length of ICU stay, days (IQR)	12(6,23)	16(10, 30)*,ǂ	10(6, 17)	9.5(6, 15)	<0.001
Length of Hospital stay, days(IQR)	22(11. 43)	36(16, 58)*	21(10, 35)	22(11, 41)	<0.001

Foot note: * p<0.05, compared to increasing group , ǂ p<0.05, compared to persistent high group

Supplementary Table 4. Effect of day 3 serum albumin on the length of stay in the ICU or hospital

	Differences in admission duration Mean (95% CI), days	P-value
**LOS at ICU, days**		
Serum albumin at D3, g/dL	0.47(-1.77, 2.71)	0.679
CRRT duration, days	1.00(0.82, 1.18)	<0.001
**LOS at hospital, days**		
Serum albumin at D3, g/dL	3.96(0.20,7.89)	0.049
Age, year	-0.16(-0.29, -0.02)	0.023
CRRT duration, days	0.92(0.62, 1.22)	<0.001

Final model: adjusted with age, SOFA score, and CRRT duration

Abbreviations: ICU, intensive care unit; CRRT, continuous renal replacement therapy; SOFA, sequential organ failure assessment; LOS, length of stay

Supplementary Table 5. Changes in fluid balance during CRRT by the serum albumin change pattern

	Persistent Low (N=299)	Increasing (N=85)	Decreasing (N=195)	Persistent High (N=214)
Total input, L	24.8±43.9	36.9±37.8	20.0±21.9	22.8±27.3
Total output, L	19.8±21.5	34.3±36.1	17.4±19.3	22.9±26.9
Total fluid balance, L	3.2±6.0	2.7±6.3	2.5±6.8	0±6.3
Changes in volume status per bodyweight. %/kg	5.6±9.5	3.2±9.2	3.2±10.2	-0.2±9.2
Volume gain≥2%/kg	20.3	27.0	22.7	43.2
Volume loss≥2%/kg	58.6	50.0	57.1	31.7
